# Improvement of Diagnostics in NSCLC Patients with *MET* Exon 14 Mutations Using Complementary DNA/RNA-NGS and Identification of Two Novel Exonic Splicing Mutations

**DOI:** 10.3390/ijms27010106

**Published:** 2025-12-22

**Authors:** Edyta Maria Urbanska, Thomas Koed Doktor, Linea Cecilie Melchior, Eva Stampe Petersson, Jens Benn Sørensen, Eric Santoni-Rugiu, Brage Storstein Andresen, Morten Grauslund

**Affiliations:** 1 Department of Oncology, Rigshospitalet, Copenhagen University Hospital, 2100 Copenhagen, Denmark; jens.benn.soerensen@regionh.dk; 2 Department of Biochemistry and Molecular Biology, University of Southern Denmark, 5230 Odense, Denmark; thomaskd@bmb.sdu.dk (T.K.D.); bragea@bmb.sdu.dk (B.S.A.); 3 Villum Center for Bioanalytical Sciences, University of Southern Denmark, 5230 Odense, Denmark; 4 Department of Pathology, Rigshospitalet, Copenhagen University Hospital, 2100 Copenhagen, Denmark; linea.cecilie.melchior@regionh.dk (L.C.M.); eva.stampe.petersson@regionh.dk (E.S.P.); eric.santoni-rugiu.02@regionh.dk (E.S.-R.); morten.grauslund@regionh.dk (M.G.); 5 Department of Clinical Medicine, University of Copenhagen, 2200 Copenhagen, Denmark

**Keywords:** *MET* exon 14 skipping, DNA/RNA-NGS, exonic mutations, MaxEntScan, SpliceTransformer, NSCLC

## Abstract

*MET* exon 14 (*MET*ex14) skipping mutations differ from other non-small cell lung cancer (NSCLC) genomic biomarkers as they result in aberrantly spliced *MET* transcripts and increased MET-signaling. However, the most accurate method for their detection remains debated. We conducted a retrospective study of previously identified *MET*ex14 skipping NSCLC samples by using different, commercially available, diagnostic targeted DNA- /RNA-Next-Generation Sequencing (NGS) panels. We primarily used small DNA-NGS panels covering the 5′ splice site of *MET*ex14 and supplemented by targeted RNA sequencing for selected cases. Using this approach, we identified <0.2% patients with *MET*ex14 mutations. Due to this low frequency, we validated and introduced complementary NGS testing using combined DNA/RNA-panels. This resulted in an increased number of *MET*ex14-positive patients (3.5%) and allowed us to identify *MET*ex14 skipping transcripts. Collectively, data from our cohort (n = 34) demonstrated that optimal diagnostics of *MET*ex14 variants require a complementary DNA-NGS performed with targeted panels covering both *MET*ex14 splice sites, and RNA-NGS. Consequently, we propose a new workflow for interpretation of concordant and discordant findings in *MET*ex14 detection. Finally, the potential of DNA-identified *MET*ex14 variants to cause aberrant splicing was in silico assessed by the MaxEntScan tool, providing a quantitative approach to splicing disruption. Interestingly, we also identified two novel variants located inside *MET*ex14, which also produced the *METex14* skipping transcript despite being located outside the canonical splice sites. The altered binding site resulting from these exonic mutations was in silico determined by SpliceTransformer.

## 1. Introduction

The N-methyl-N’-nitro-N-nitrosoguanidine (MNNG) transforming gene, *MET*, encodes the hepatocyte growth factor (HGF) receptor, MET, which plays a fundamental role in regulating cell differentiation, migration, and growth [1]. Pathogenic activation of MET is a well-defined oncogenic driver in many types of cancers [2]. *MET* exon 14 (*MET*ex14) skipping variants occur in 0.6–4% of non-small cell lung cancer (NSCLC), adenocarcinomas [2,3], and belong to the group of molecular biomarkers whose testing is recommended in international guidelines [4,5]. Importantly, NSCLC with *MET*ex14 skipping is associated with poor prognosis, as the median overall survival for patients with these mutations when treated with MET tyrosine kinase inhibitors (MET-TKIs) is 24.6 months, while for those not receiving MET-TKIs it is 8.1 months [6]. This demonstrates a strong therapeutic potential in detecting and targeting *MET*ex14 skipping mutations. 

The activity of the MET receptor is a result of a dynamic balance between activation by its ligand, HGF, and degradation by the E3 ubiquitin ligase, Casitas B-lineage lymphoma (CBL) protein [7]. *MET*ex14 codes a part of the regulatory region in the juxtamembrane domain (JM) that is the docking site for CBL in the MET protein [8]. Consequently, the lack of JM in cells with *MET*ex14 leads to an increased steady-state level of MET protein [9]. Additionally, rare mutations in JM, such as amino acid Y1003 (ubiquitin ligase binding site), D1002 (caspase cleavage site), or S985 (phosphorylation site), may reduce degradation of the MET receptor mimicking a *MET*ex14 skipping without aberrant splicing [10]. All these alterations enhance the pleiotropic MET signaling, which drives cancer progression via activation of numerous signaling mechanisms like activation of the RAS-MAP kinase pathway, SMAD2/3-signaling independent of TGF-β, and AKT-driven invasive growth [11,12,13].

In eukaryotic cells, mRNA splicing is conducted by the spliceosome, a large RNA-protein complex, which recognizes the splice donor (SD) site at the 5′ end of the intron and the splice acceptor (SA) site at the 3′ end. Near the SA site lies the polypyrimidine tract (PPT), which helps recruit splicing factors. Upstream of this tract is the branch point (BP), an adenosine that initiates the lariat formation during intron removal. Together, these elements ensure precise exon joining and intron excision [14,15]. The SD site consists of the 3 last nucleotides of the exon and the first 8 nucleotides of the intron, and the SA site consists of the last 20 nucleotides of the intron and the first 3 nucleotides of the exon [16,17,18,19,20]. Most exons, including *MET*ex14, have their 3′SA and 5′SD with sequence AG (corresponding to the end of an intron) and GT (corresponding to the beginning of an intron), respectively, known as canonical splice sites [19]. Regulation of mRNA splicing leading to alternative splicing is regulated by complex interactions between the spliceosome and splicing regulatory proteins, which bind *cis*-acting splicing regulatory elements (SREs) like exonic splicing enhancers and silencers (ESEs and ESSs, respectively) and intronic splicing enhancers and silencers (ISEs and ISSs, respectively). An SRE mutation is defined as any nucleotide change within an exon (or sometimes intron) that creates, disrupts, or alters the strength of an SRE motif, thereby influencing splicing. These motifs recruit splicing factors such as SR and hnRNP proteins. The exonic mutations may affect both SREs—ESEs and ESSs. ESEs promote exon inclusion, and ESSs inhibit splicing. However, the activity of SREs depends on position (in one region they may act as ESEs and in another region as ESSs) and gene expression affecting the regulatory effect. Exonic mutations can disrupt ESE/ESS motifs causing mis-splicing: exon skipping or activation the cryptic splice sites. About 25–27% of exons are vulnerable to splicing disruption by exonic mutations [21]. However, the role of exonic mutations has not been described for *MET*ex14. In summary, SRE mutations are defined by location (outside the canonical splice sites), motif disruption, and functional consequence. Using in silico prediction (ESEfinder, SpliceTransformer) we can localize the altered binding site.

Aberrant splicing is associated with the pathogenesis of different diseases, including NSCLC with skipping mutations in *MET*ex14 [2,8].

A growing number of somatic DNA splice site mutations leading to *MET*ex14 skipping have been identified [2,22]. The majority of these *MET*ex14 splice site mutations are base substitutions/small deletions at the SD, BS, PPT, and SA sites [22]. The mechanism of *MET* mRNA splicing is also clinically important for explaining the outcome of MET-TKI treatment. Since splice site recognition depends on matching the splicing consensus, alterations in splice sites may result in alternative splicing [17]. In particular, intolerable mismatches due to disrupted sequences lead to aberrant splicing. Exonic mutations may also cause exon skipping by disrupting exonic splicing enhancers or by increasing the strength of exon splicing silencers [16,17,21].

Next-generation sequencing (NGS) is a preferred method for testing molecular driver alterations in NSCLC. Most clinical laboratories use NGS panels of onco- and tumor suppressor genes for detecting DNA single nucleotide variants (SNVs), small deletions, splice site mutations, copy number variants (CNVs), and gene fusions. Both DNA-based and RNA-based NGS can be used to detect *MET*ex14 skipping mutations and transcripts. The former detects splice site mutations in the splice site regions that are predicted to lead to *MET*ex14 skipping, whereas RNA-NGS detects the direct fusion of *MET* exon 13 and exon 15 transcripts [6]. 

In our study, we investigated whether combining DNA-based and RNA-based NGS analyses would improve sensitivity and specificity of *MET*ex14 skipping detection in NSCLC samples. Furthermore, we compared targeted DNA-NGS panels commonly used in diagnostics of NSCLC and analyzed how they may perform on the detection of *MET*ex14 splice site mutations. We present data based on our own approach to *MET*ex14 skipping detection, which supports complementary DNA- and RNA-based NGS in clinical routine.

Since NSCLC is a disease often driven by subclonal alterations, a quantitative approach to *MET*ex14 variants might help to better define their impact on the level of protein lacking sequence encoded by exon 14. Therefore, we explored this aspect by using the bioinformatic tool MaxEntScan (MES) to predict the effects of sequence variations on splicing signals [23]. Our cohort was analyzed to investigate the potential relationship between MES scores and genomic localization of detected *MET*ex14 splice site variants, alongside clinical features such as age, gender, histological diagnosis, smoking history, and presence of brain metastases.

## 2. Results

### 2.1. Identification of METex14 Positive NSCLC Patients

**Figure 1:**
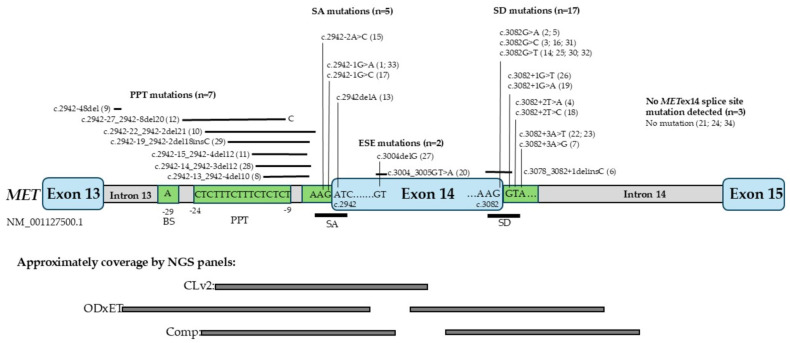
Localization of detected *MET*ex14 mutations and coverage of used NGS panels. BS: branch site, PPT: poly-pyrimidine tract, SA: 3’splice acceptor site, SD: 5’splice donor site, ESE: exonic splicing element, CLv2: AmpliSeq Colon and Lung Cancer Research Panel v2, ODxET: Oncomine Dx Express Test, Comp: Oncomine Comprehensive v3.

**Figure 2:**
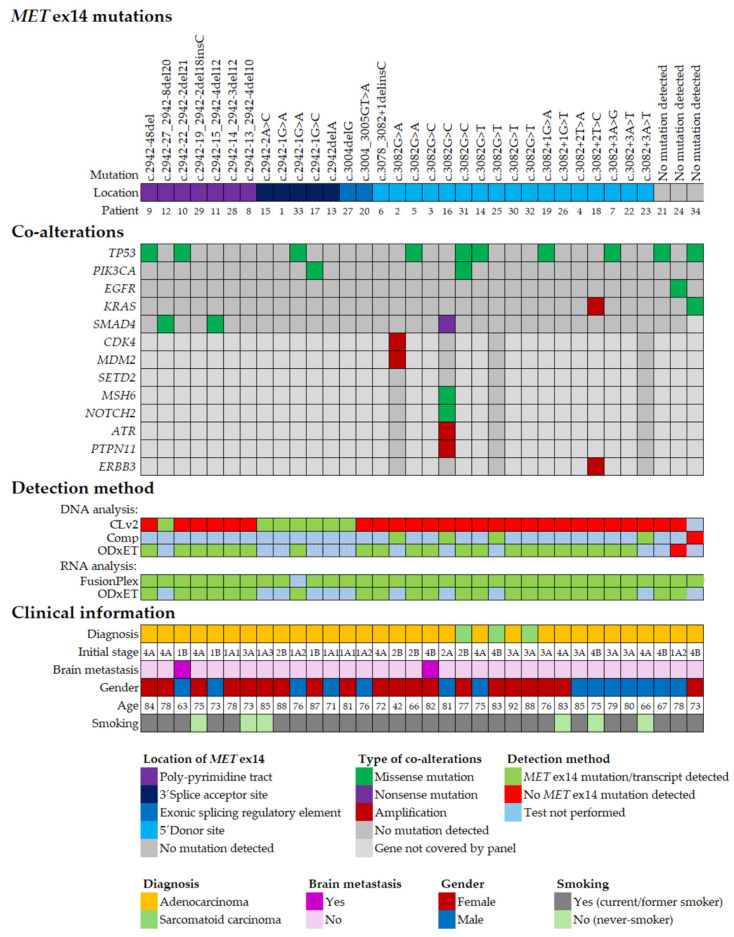
Molecular and clinical features of the 34 patients harboring somatic *MET*ex14 alterations. *MET*ex14 mutations: mutation detected in Branch Site (BS) and polypyrimidine tract (PPT) (purple), 3’splice acceptor site (dark blue), exonic splicing regulatory element (ESRE) (blue), 5’ splice donor site (light blue), and no mutation detected (grey). Co-alterations: missense mutation (dark green), nonsense mutation (purple), amplification (dark red), no mutation detected (dark grey), and no mutation detected (light grey). Detection method: *MET*ex14 mutation/transcript detected (green), *MET*ex14 mutation not detected (red), and test not performed (light blue). Clinical information: diagnosis; adenocarcinoma (yellow) and sarcomatoid carcinoma (light green), brain metastasis; yes (brown) and no (light blue), gender: female (brown) and male (blue), smoking; current/former smoker (grey) and never-smoker (light green).

Between January 2018 and August 2023, routine molecular testing of NSCLC using mainly the AmpliSeq Colon and Lung Cancer Research Panel v2 (CLv2) identified seven cases with *MET*ex14 splice site mutations among 5000 tested non-squamous NSCLC cases. In three cases, an additional NGS analysis was performed using the Oncomine Comprehensive v3 panel (Comp), and four additional cases were identified with *MET*ex14 splice site mutations. During the same period, NGS-RNA testing was conducted with the Archer FusionPlex Lung Panel v1.0 on approximately 350 selected cases (e.g., younger patients, non-smokers, or those with a detected *MET*ex14 splice site mutation), identifying 30 *MET*ex14 skipping transcript-positive cases with adenocarcinoma histology and three cases with sarcomatoid carcinoma histology The observed frequency of *MET*ex14 skipping mutation-positive patients identified by DNA-NGS was much lower (approximately 0.15%) than the reported frequency of 0.6–4% in NSCLC patients [2,3]. A possible explanation for the low incidence of *MET*ex14 skipping variants detected with the CLv2 panel is its limited amplicon coverage of *MET*ex14, as it includes only the SA site and a part of the PPT region (Figure 1). To improve diagnostic sensitivity, we analyzed 12 cases with the Comp NGS panel with confirmed *MET*ex14 skipping transcripts, but no splice site mutations were found by the CLv2 panel. In 10 of these cases, splice site mutations not covered by the CLv2 panel were detected (Figure 1 and Figure 2, Supplementary Tables S1 and S2). In two patients (cases 21 and 24), no *MET*ex14 splice site mutation was identified, likely due to larger genomic structural variants [2]. In August 2023, the combined DNA/RNA-NGS panel Oncomine Dx Express Test (ODxET) was clinically implemented at our site as the standard procedure for molecular diagnostic testing of all pulmonary adenocarcinomas. Among the first 519 patient samples analyzed using this method, 19 *MET*ex14-positive cases (3.3%) were identified, all showing both a *MET*ex14 splice site mutation and exon 14 skipping transcripts.

### 2.2. Subtypes of METex14 Mutations

To gain a deeper understanding of the diversity of *MET*ex14 splice site mutations leading to *MET*ex14-skipping transcript, we analyzed the subtypes of *MET* mutations based on their location in the gene. In total, using these three different DNA-NGS panels, we identified 17 samples with *MET* mutations causing aberrant splicing, which were localized in the 5′ SD site, seven cases with mutations in the PPT region located in intron 13, and five patients with mutations of the SA site. Lastly, in two cases, a potential ESE was affected by previously unreported mutations localized in the middle of exon 14 (Figure 1 and Figure 2). 

The two most frequently identified splice site mutations were c.3082G>C and c.3082G>T, present in three and four samples, respectively. These two mutations were also among the most common splice site mutations identified by Kim et al. [22]. All mutations identified in the SA- and SD sites were described in previous studies [2,22]. As formerly reported [8,22], we also observed a wide distribution of deletions within the PPT region in seven affected cases. In one instance (case 9), we identified a 1 bp deletion located 48 base pairs upstream of the SA element. The impact of this short deletion on *MET*ex14 splicing is unclear.

### 2.3. Co-Occurring Genomic Alterations in METex14 Positive NSCLC

We analyzed whether the *MET*ex14 positive samples in our cohort harbored alterations in other relevant genes. We identified co-existing genetic alterations in 53% (18/34) of *MET*ex14-skipping positive patients (Figure 2, Supplementary Table S3). Co-mutations of the tumor-suppressor gene *TP53* were the most frequent co-alteration, occurring in 10/34 patients (29%). Two patients with *MET*ex14 alterations (case 24 and 34) also harbored concurrent mutations in the oncogenic drivers, *EGFR* and *KRAS*, respectively. Notably, in both cases, *MET*ex14 mutations were detectable only at the RNA transcript level, with no corresponding DNA splice site mutations identified. The patient with the *EGFR* co-mutation exhibited the well-known pathogenic p.L858R substitution in the tyrosine kinase domain of EGFR and, because of stage II disease, underwent curative surgery. In this case, *MET*ex14 transcripts were present at low levels, as confirmed by two independent RNA-based assays (FusionPlex and OdxET). Since no *MET*ex14 DNA splice site mutations were detected, we cannot rule out that the observed *MET*ex14 transcript results from background splicing [14,16]. On the other hand, the patient with *KRAS* p.G12V mutation had a high level of *MET*ex14 transcripts. Other identified pathogenic co-alterations were mutations in *SMAD4*, *PIK3CA*, *SETD2*, *MSH6*, and *NOTCH2*, as well as amplification of *CDK4*, *MDM2*, *ATR*, and *PTPN11*. In two patients (cases 31 and 34), co-mutation of *TP53* and *PIK3CA* were found, while in one patient (case 2) the NSCLC cells harbored co-amplification of *CDK4* and *MDM2*, and in another case (no. 16) co-existing mutations in *MSH6*, *NOTCH2*, and *SMAD4* together with amplification of *ATR* and *PTPN11* genes were detected (Figure 2, Supplementary Table S3). Interestingly, no *MET* amplification was observed, which in approximately 15% of cases co-occurs with the *MET*ex14 skipping mutation; this may be explained by the small size of the patient group [8,22]. Since the gene content differs in the used NGS panels (Supplementary Table S1), not all genes were analyzed across all samples.

### 2.4. Clinical Characteristics of METex14 Positive NSCLC

Most of the patients were ≥70 years old (30/34), with co-existing comorbidities (30/34), in performance status (PS) 0–1 (32/34), females (20/34), with no spread to the central nervous system (32/34), in advanced/metastatic stage (18/34), and previous smokers (28/34) (Supplementary Table S4). The clinical characteristics of our cohort are presented in Figure 2 and are consistent with previous observations in the literature and larger cohorts [6,8,20,24,25]. There were only 5 patients treated with MET-TKI, which makes our cohort too small for reliable statistical analysis of the clinical effect of MET-TKI. Patients no 3, 4, and 18 are still being treated with MET-TKI (November 2025) and all had mutations in the SD site (Figure 2). 

### 2.5. Mutations in an Exonic Splicing Enhancer in METex14 Dependent for Splicing

**Figure 3:**
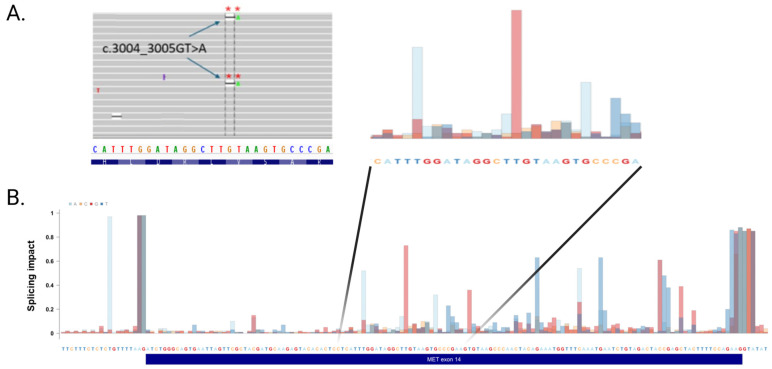
Identification of novel exonic mutations in *MET*ex14 resulting in *MET*ex14 skipping. (**A**). Visualization of exonic *MET*ex14 c.3004_3005GT>A mutation in patient case 20. (**B**). SpliceTransformer analyses of *MET*ex14. “**” represent the location of the nucleotide alterations.

Interestingly, two of the identified *MET*ex14 skipping transcript positive cases had exonic *MET*ex14 mutations (case 27: c.3004delG and case 20: c.3004_3005GT>A) involving the same position. Because these mutations both lead to a reading frame shift, which would cause nonsense-mediated decay, it was surprising that *MET*ex14 transcripts were identified by RNA-NGS testing (Figure 2), although with a reduced number of *MET*ex14 transcripts (114 and 515 reads, respectively). To investigate these two exonic mutations, we performed an analysis using the ESE finder tool (version 3.0), which identified a potential altered binding site for the serine/arginine-rich splicing factor 2 (SRSF2) (Supplementary Table S5). Furthermore, we analyzed the effect of mutation in each nucleotide in *MET*ex14 using the newly developed SpliceTransformer (SpTransformer) algorithm [26]. SpTransformer identified in silico that the SD and SA sites were most affected by point mutations (Figure 3) [27]. Furthermore, several potential *MET*ex14 exonic SREs, including nucleotide c.3003 which is located in the potential SRSF2-binding site and neighboring nucleotide c.3004 which is deleted in cases 20 and 27, were identified using ESE finder. These findings suggest that exonic mutations within *MET*ex14 may influence its splicing by disrupting SREs and thus be important in the choice of MET-TKI therapy.

### 2.6. In Silico Prediction of METex14 Splice Site Mutations

**Figure 4:**
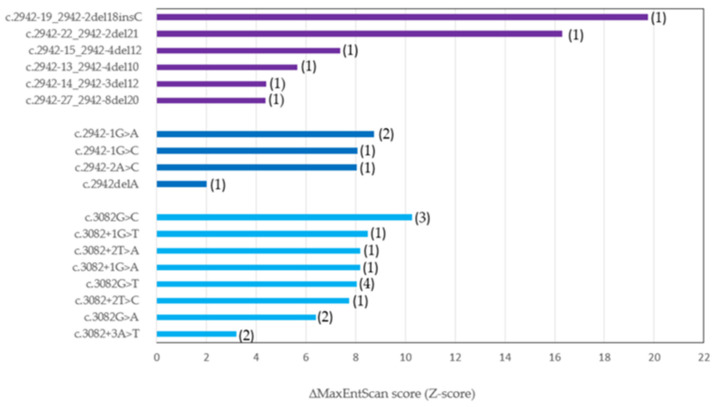
Prediction of impacts of *MET*ex14 splice site mutations evaluated as maximum entropy scores (Z-scores) tool presented difference in MaxEntScan score (MaxEntScan score for wild type sequence—MaxEntScan score for *MET*ex14 mutated sequence). Mutations with Z-scores > 0 are predicted to be pathogenic. Location of mutation: branch site and poly-pyrimidine tract (purple bar), SA site (dark blue), and SD site (light blue). The remaining *MET*ex14 splice site mutations could not be analyzed using this algorithm due to locations too distant from the SA and SD elements. The numbers in brackets 1–4 indicate the amount of patients with a specific *MET*ex14 variant.

MES relies on the “Maximum Entropy Principle” and extends beyond many earlier probabilistic models of sequence motifs, including weight matrix models and inhomogeneous Markov models [23]. Analysis of the *MET*ex14 splice sites using MES showed that *MET*ex14 contains a relatively strong SA splice site with a score of 10.86 and a relatively weak SD splice site with a score of 7.84. Two patients with the strongest effect of *MET*ex14 mutations had relatively large deletions of the 5’ parts of the PPT element (case 10 and 29). The lowest MES score difference was observed in the SA mutation c.2942delA (score: 2.02) and the SD mutation c.3082+3A>T (score: 3.20), which might be less severe and only partially result in the expression of *MET*ex14 transcript. The MES analysis of the *MET* c.2942-48del (case 9) mutation could not be performed due to the mutation’s location too distant to the SA and SD sites (Figure 4, Supplementary Table S6).

Our MES analysis showed the highest Z-scores for identified splice site mutations such as c.2942-1G>A and c.3082+2T>A and the lowest Z-scores for mutations in less conserved nucleotide positions like c.2942delA and c.3082+3A>T. 

## 3. Discussion

Reliable detection of potentially pathogenic mutations in *MET*ex14 is critical for identifying NSCLC patients who will benefit from MET-TKIs. NGS is a frequently used method for detecting genetic alterations in NSCLC, including *MET*ex14 skipping variants. Advantages of utilizing NGS-technology include the ability to analyze multiple biomarkers simultaneously within a single assay with acceptable sensitivity (approximately 5% mutated allele). This approach conserves both time and tumor tissue when compared to sequential testing of individual biomarkers [28]. The phenomenon of *MET*ex14 skipping was first identified in 1994 as an alternative splicing variant in cDNA from normal mouse embryos, without any associated mutations affecting splicing consensus sequences [29]. *MET*ex14 skipping was first identified in NSCLC tissue in 2005, resulting from a somatic 141 bp deletion that led to the skipping of exon 14 [30]. *MET*ex14 splice site variants occurring in NSCLC are now counted in hundreds [22,24,25]. Certain *MET*ex14 splice sites variants such as c.3082+1G>T and c.3082+1G>A are already well-defined in genomic databases like the Catalogue of Somatic Mutations in Cancer (COSMIC), the Oncology Knowledge Base (OncoKB), and cBio Cancer Genomics Portal (cBioPortal), in terms of their impact on splicing [31,32,33]. 

In our small cohort (n = 34), the clinical and molecular data are also comparable with the largest to date reported cohort of 1592 NSCLC patients with *MET*ex14 skipping variants detected by comprehensive hybrid capture-based genomic profiling (69,219 NSCLC samples profiled) [25]. This study confirmed the comparable frequency of this alteration (2.3% versus 3.5% in our cohort), more frequent prevalence in older populations with smoking history, and co-existing mutations in tumor suppressor genes like *TP53* and *MDM2*. Additionally, the feasibility of detecting *MET*ex14 in plasma (n = 134) was demonstrated to expand the applications of plasma as the sole source for testing.

To ensure the most reliable detection of *MET*ex14 variants, sufficient coverage on both splice sites should be granted, and this, as illustrated in Figure 1, varies between different DNA-NGS panels. It is important to use an NGS panel covering relevant sequences of both the SA and the SD sites, as well as the BS and PPT elements of *MET*ex14 [34]. Indeed, the CLv2 panel only covers the SS element of *MET*ex14 as well as parts of *MET* intron 13 and exon 14, which might explain why only approximately 10% of the possible *MET*ex14 splice site mutations can be detected using this panel [34]. In addition, not all *METe*x14 skipping mutations can be reliably detected by DNA-based NGS alone, as larger genomic rearrangements or mutations within *MET*ex14 exonic SRE elements may be challenging to identify with certain DNA-NGS panels, particularly older panels such as the CLv2 panel. Therefore, to detect *MET*ex14- and other gene fusion-positive patients, the most optimal diagnostic approach is to use combined DNA- and RNA-NGS [35,36,37,38].

**Figure 5:**
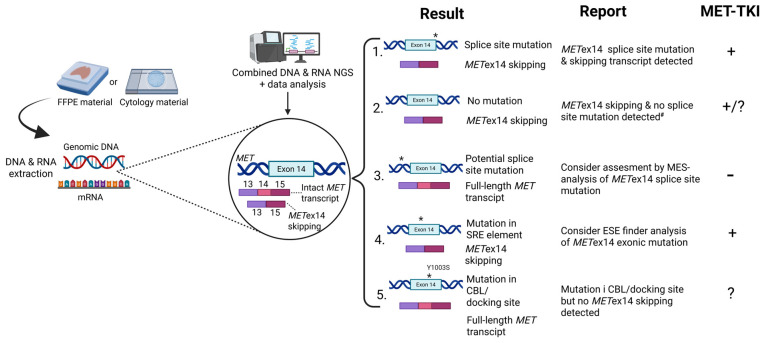
Proposed workflow for diagnostics and interpretation of complementary DNA- and RNA-NGS testing results in NSCLC patients with *MET*ex14 mutations and the clinical implications for using MET-TKI. “*” represents mutation in or outside exon 14. ^#^ Discordance in scenario 2 between the presence of the shorter *MET* transcript and the lack of a *MET* mutation, which provoked aberrant splicing. It requires an individual approach depending on the number of reads and the NGS analysis used. “+” supports using MET-TKI; “−” does not support using MET-TKI; “?” using MET-TKI is uncertain.

Therefore, optimal insight into this biomarker utilizing sequencing of DNA and RNA is needed to achieve a reliable detection supported by information from both materials. As shown in Figure 5, we present an optimized diagnostic workflow that has already been implemented at our institution. This diagnostic workflow reflects synergy of complementary DNA- and RNA-testing and allows optimal interpretation of both results, leading to a more sensitive approach, and provides direct access to an aberrant splicing product.

The five main diagnostic outcomes from combined DNA and RNA sequencing are outlined in Figure 5. The most common scenario (1) involves the detection of *MET*ex14 splice site mutations leading to exon skipping. When both mutations causing the exon 14 skipping and a shorter transcript are present, the concordant results support using MET-TKI. In the second instance (2), *MET*ex14 skipping is detected in the absence of identifiable splice site mutations within exon 14. Accurate quantification of *MET*ex14 transcripts is critical in these cases, as the presence of a shorter transcript is sufficient for producing abnormal protein, inaccessible for ubiquitination, and therefore can serve as a target for MET-TKI. Amplicon-based NGS analyses have occasionally demonstrated low-level expression of *MET*ex14 skipping. However, it is still uncertain whether this reflects physiological background expression or results from assay-related artifacts. Given that transcript detection is influenced by assay design and sequencing depth, it is essential for each laboratory to define the assay-specific thresholds to ensure reliable interpretation within the diagnostic context. In cases exhibiting high *MET*ex14 transcript expression, the most probable underlying mechanism is large-scale chromosomal rearrangements affecting *MET* exon 14 splicing, which are not detectable by the employed NGS panel due to its limited genomic coverage [34,39,40]. In the third scenario (3), where a potential *MET*ex14 splice site mutation is identified, but no corresponding exon 14 skipping transcript is observed, we recommend conducting a bioinformatic evaluation of the variant using, e.g., MaxEntScan. This tool aims to assess the splice site’s strength and to indicate whether the identified mutation has a functional impact on splicing. In this case, the usage of MET-TKI is unlikely to have an effect since MET protein stability should not be affected [41]. The fourth case (4) involves the identification of an exonic mutation within *MET*ex14 accompanied by exon 14 skipping, but without any detectable splice site mutation. In such instances, it is valuable to further investigate whether the exonic variant is located within an ESE motif, as disruption of ESE elements could potentially influence *MET*ex14 splicing. Notably, synonymous exonic mutations not leading to amino acid sequence change (i.e., silent mutations) are often filtered out during bioinformatic analyses, although they may affect ESE elements and consequently affect exon splicing. This rare scenario reflects the vulnerability of exon 14, which is its unique feature inducing aberrant splicing, while canonical splice sites are undisturbed. The utilization of MET-TKI is legitimized here due to the presence of *MET*ex14 skipping. The fifth scenario (5) involves the domain of *MET*ex14 where CBL binds, which can also result in sustained MET receptor activity. Importantly, mutations affecting amino acid residue Y1003—a critical site for CBL-mediated recruitment and subsequent ubiquitination of MET—can deregulate MET signaling. These mutations phenocopy the functional effects of *MET*ex14 skipping by impairing receptor downregulation, although they do not alter MET splicing [7,10]. Additionally, studies from cell lines have proved that altered CBL activity may also cause MET receptor activity, with CBL wildtype cells showing lower MET expression than CBL mutant cells. Since ubiquitination of MET was also decreased in CBL-mutant cells compared to CBL-wildtype cells, CBL status was proposed to be considered a potential positive indicator for MET-targeted therapy in NSCLC [7,42]. However, despite anecdotal case reports having described the clinical effect of the MET-TKI, Crizotinib, in NSCLC patients with MET Y1003S mutation [43,44], the efficacy of MET-TKI in this scenario remains uncertain [45].

Our workflow demonstrated in Figure 5 addresses the issue of discordance between DNA- and RNA-NGS and is primarily built on our experience with amplicon-based NGS. Hybrid-capture assay is an alternative technique that may also be considered in situations where DNA-NGS did not identify a *MET*ex14 mutation but a *MET*ex14 skipping transcript is present. In such cases, *MET*ex14 skipping can be caused by large structured chromosomal alterations, which cannot be detected by amplicon-based NGS. However, it requires more DNA and RNA input, which can be challenging with scarce diagnostic materials. Furthermore, it is a more costly and time-consuming approach [25,40].

The impact of each *MET*ex14 variant on splicing may play an important role in understanding this disease. The question is whether it is possible that each *MET*ex14 variant can be quantitatively defined in a way that determines its role as a driver alteration in a particular patient. To address the question of quantitative assessment of *MET*ex14 skipping variants, different approaches, like variant allele frequency (VAF) or number of RNA reads, may be taken into consideration. VAF means the percentage of reads of a given variant in relation to all reads in this position, and RNA reads give numbers of a given *MET* transcript. Despite these quantitative features that may inform about the measurable presence of a given *MET*ex14 variant, they all depend on spatial and temporal conditions determining the specific biopsy of the tumor at the given time. As NSCLC is an evolving disease, these conditions are dynamic and cannot be regarded as independent and robust factors, implying that the individual *MET*ex14 variant may or may not function as a strong driver in this disease. 

Another approach may be in silico analysis providing a numerical assessment of a variant impact, which may be calculated by the MES tool. MES is a bioinformatics tool used to predict the effects of sequence variations on splicing signals by modeling the sequences of short sequence motifs involved in RNA splicing, and accounts for non-adjacent and adjacent dependencies between positions. We have calculated the impact of *MET*ex14 variants identified in our cohort by MES, and the results are presented in Figure 4. The varying levels of *MET*ex14 variant expression reflect differences in their functional impact—specifically, their capacity to drive a MET-dependent phenotype in NSCLC. The MES value determines the extent of MET-dependent signaling and contributes to disease heterogeneity. The impact of the individual *MET* variants and influence of co-existing alterations both create the individual molecular picture of each patient with the *MET*ex14 skipping variant. Then, these two factors: the MES score of *MET* variants and co-alterations may affect the response to targeted therapy with MET-TKIs. The observations from clinical studies show that the best response rates to the current available MET-TKIs reach about 70% in treatment-naïve patients [46]. It shows us that there might be several subgroups among NSCLC with *MET*ex14 skipping defined by different strengths of *MET*ex14 variants on aberrant splicing and MET signaling. Furthermore, co-existing molecular alterations may also have an impact on MET-TKI response [22,47,48]. Altogether, these factors may define individual subgroups of NSCLC patients with *MET*ex14 splice site variants. 

Bioinformatics tools such as MES and SpTransformer may be used as complementary algorithms to prioritize variants [23,26]. When a variant is located within the splice site region, MES can be used to directly assess the effect on splice site strength (Figure 4), while SpTransformer allows for the estimation of the overall splicing impacts of variants both within and outside the splice site region (Figure 3). Variants flagged by SpTransformer can then be further analyzed with tools like MES to determine whether they alter splicing by creating a novel splice site, or by disturbing the balance between splicing enhancers and silencers.

Despite promising results of variant interpretation in silico by using DNA-NGS data, especially regarding *MET* variants not interrupting splice sites, the access to bioinformaticians may be a significant hurdle in the routine practice [49]. Furthermore, employing several recently developed machine learning-based algorithms, demonstrating a high potency to predict splicing and to classify transcript variants of *MET*ex14, may also necessitate collaboration with data scientists, who are still difficult to access in the real-world clinical diagnostics [50,51]. Finally, computational-based evidence predicting impact on skipping may not always be acceptable to categorize a new potentially (likely) pathogenic variant as pathogenic, and further functional studies may be required [52]. By using this approach, two *MET*ex14 splice site variants located outside the canonical GT/AG have recently been identified by integrating the combination of in silico prediction, RT-PCR, with Sanger sequencing and adopted to a laboratory standard procedure as routine practice [53].

## 4. Materials and Methods

### 4.1. Patient Cohort and Molecular Diagnostic Testing

We identified 34 NSCLC patient samples harboring *MET*ex14 variants as DNA splice site mutations and/or *MET*ex14 RNA transcript, from the targeted NGS data of the routine molecular testing of NSCLC-patients at the Department of Pathology, Rigshospitalet, Denmark, between January 2018 and August 2023. In this period, we used different DNA-NGS panels as a core diagnostic approach, and RNA-NGS as a supplementary and gradually implemented diagnostic tool. Molecular diagnostic NGS analyses of lung cancer patients were carried out initially (January 2018–August 2023) using the DNA-based AmpliSeq Colon and Lung Cancer Research Panel v2 (CLv2) (Thermo Fisher Scientific, Roskilde, Denmark) and in certain cases supplemented with broader NGS tests by using the DNA-based part of the Oncomine Comprehensive Panel v3 (Comp) (Thermo Fisher Scientific. Roskilde, Denmark), and/or the RNA-based FusionPlex Lung Panel v1.0 (FusionPlex) (Archer, Boulder, CO, USA). In August 2023, the NSCLC molecular diagnostic testing was substituted with combined DNA and RNA testing using the Oncomine Dx Express Test (ODxET) panel (Thermo Fisher Scientific, Roskilde, Denmark). Detailed gene lists of the NGS panels are provided in Supplementary Table S1. Samples without *MET*ex14 transcript and *MET*ex14 splice site mutation were retrospectively re-sequenced as part of this study using the ODxET panel. The Comp and FusionPlex panels were sequenced on an Ion GeneStudio™ S5 System (Thermo Fisher Scientific, Roskilde, Denmark) and the ODxET panel was sequenced on a Genexus™ Integrated Sequencer system (Thermo Fisher Scientific, Roskilde, Denmark). Both instruments were used for the CLv2 panel.

### 4.2. Genomic Profiling by Next-Generation Sequencing

Genomic DNA was isolated from tissue sections of formalin-fixed paraffin-embedded (FPPE) tumor resections or core needle biopsies (n = 18) using the One-tube FFPE extraction method [54] or from cytological tumor fine-needle aspirates (n = 16), (Supplementary Table S3) using Maxwell RSC DNA Blood kit (Promega, Madison, WI, USA). RNA was isolated from both materials using the Maxwell RSC RNA FFPE kit.

### 4.3. Bioinformatic Analysis

Splice site mutations were in silico analyzed using MES (https://github.com/Congenica/maxentscan (accessed on 24 November 2025)) [23]. Prediction of exonic splicing enhancers (ESEs) was performed by using ESE finder 3.0 (https://esefinder.ahc.umn.edu/cgi-bin/tools/ESE3/esefinder.cgi (accessed on 24 November 2025)) [55] and SpTransformer (http://tools.shenlab-genomics.org/tools/SpTransformer (accessed on 24 November 2025)) [26]. All bioinformatic analyses were performed using *MET* reference transcript sequence NM_001127500.1 (https://www.ncbi.nlm.nih.gov/nuccore/NM_001127500.3/ (accessed on 24 November 2025)).

## 5. Conclusions

This study has demonstrated:

- When using DNA-NGS technology to detect *MET*ex14 skipping variants, it is important to note that different panels, such as CLv2, ODxET, and Comp, are designed to capture splice sites mutations in specific regions of exon 14.
- Complementary DNA- and RNG-NGS are needed for optimal detection of *MET*ex14 skipping in real-world NSCLC patients.
- The presence of the aberrant *MET* transcript is the most predictive biomarker for using MET-TKIs.
- Bioinformatics tools such as MES and SpTransformer provide additional information regarding impact of each *MET*ex14 mutation on aberrant splicing and the altered binding site, respectively.
- Two novel exonic mutations are also capable of causing abnormal splicing of *MET*ex14, in addition to variants localized in canonical splice sites.

## Data Availability

The original contributions presented in this study are included in the article/Supplementary Materials. Further inquiries can be directed to the corresponding author.

## Supplementary Materials

The following supporting information can be downloaded at: https://www.mdpi.com/article/10.3390/ijms27010106/s1.
